# Association of Adding Salt to Foods and Potassium Intake with Incident Atrial Fibrillation in the UK Biobank Study

**DOI:** 10.31083/j.rcm2509332

**Published:** 2024-09-19

**Authors:** Yoon Jung Park, Pil-Sung Yang, Bo Eun Park, Jong Sung Park, Eunsun Jang, Daehoon Kim, Hong Nyun Kim, Namkyun Kim, Jang Hoon Lee, Yongkeun Cho, Jung-Hoon Sung, Boyoung Joung

**Affiliations:** ^1^Division of Cardiology, Department of Internal Medicine, Kyungpook National University Chilgok Hospital, 41404 Daegu, Republic of Korea; ^2^Department of Internal Medicine, School of Medicine, Kyungpook National University, 41944 Daegu, Republic of Korea; ^3^Department of Cardiology, CHA Bundang Medical Center, CHA University, 13496 Seongnam, Republic of Korea; ^4^Division of Cardiology, Department of Internal Medicine, Kyungpook National University Hospital, 41944 Daegu, Republic of Korea; ^5^Division of Cardiology, Department of Internal Medicine, Severance Cardiovascular Hospital, Yonsei University College of Medicine, 03722 Seoul, Republic of Korea

**Keywords:** sodium, potassium, atrial fibrillation, mortality

## Abstract

**Background:**

High sodium and low potassium consumption are related to hypertension and cardiovascular disease. We aimed to determine the relationship between the frequency of salt addition and potassium consumption with the risk of new-onset atrial fibrillation (AF).

**Methods:**

Our study used the UK Biobank cohort, which included over 500,000 individuals enrolled from the United Kingdom between 2006 and 2010. This study involved 416,868 participants who filled out the dietary recall regarding the frequency of salt addition.

**Results:**

During follow-up, 19,164 (4.6%) developed AF. The incidence of new-onset AF was increased based on the frequency of salt addition (never/rarely 3.83; always 4.72 per 1000 person-years). Compared with the group that never/rarely added salt, those adding salt always were at significantly higher risk of incident AF after adjusting for multiple variables (hazard ratio (HR) 1.15; 95% confidence interval (CI) 1.06–1.24), and additional adjustment of dietary and total energy consumption (HR 1.37; 95% CI 1.08–1.73). In the subgroup analysis, the risk of AF incident according to the frequency of salt addition significantly increased in low urine potassium levels compared to high (*p* for interaction = 0.046). In the subgroup analysis for AF patients, higher salt addition frequency was related to increased all-cause mortality.

**Conclusions:**

Our study demonstrated that adding salt to foods more frequently increases the risk of incident AF, even after adjusting for dietary and total energy consumption. In the high urine potassium group, the impact of high sodium consumption on incident AF was attenuated.

## 1. Introduction

Atrial fibrillation (AF) is associated with an elevated risk of ischemic stroke, cognitive dysfunction, heart failure (HF), and all-cause mortality [1, 2, 3, 4, 5]. Among the various factors leading to the development of AF, hypertension, ischemic heart disease, and HF are major risk factors for the onset of AF [6, 7, 8].

High sodium consumption substantially contributes to blood pressure [9, 10] and is regarded as a risk factor for cardiovascular disease (CVD) [11, 12, 13]. Several studies showed that increased dietary sodium consumption was related to increased blood pressure [10, 14]. In a meta-analysis study, increasing sodium consumption was associated with an increased risk of hypertension [15]. Additionally, other studies showed that increased sodium consumption is related to an increased risk of CVD [12, 16]. Moreover, among prehypertensive individuals, reduced sodium consumption may also reduce the risk of CVD [17, 18]. However, several studies have presented contradictory findings concerning the correlation between sodium consumption and the risk of AF incidents. Whereas one study indicated a U-shape correlation between sodium consumption and risk of AF incident [19], another study revealed a positive relationship between high sodium consumption and elevated risk of AF incident [20]. Other factors, such as potassium consumption, may modify the correlation between sodium consumption and the risk of other outcomes. It was reported that high sodium-to-potassium ratio and low potassium excretion were related to an elevated risk of CVD [16]. In the International Study of Salt and Blood Pressure study, a high urinary potassium-to-sodium ratio was related to reduced blood pressure. This ratio showed a statistically more robust relationship with blood pressure than the excretion of sodium or potassium alone [18]. However, no studies have demonstrated a correlation between potassium consumption and AF incidents.

While investigating the relationship between sodium consumption and other clinical outcomes, the assessment of sodium consumption has yet to be standardized due to methodological limitations. Estimated sodium consumption includes 24-hour urinary sodium levels calculated from spot urine measurement samples [9, 14], direct measurement of 24-hour urinary sodium levels [16], and dietary assessment concerning the frequency of salt addition [12, 21]. As a marker of sodium consumption, 24-hour urinary sodium levels were used in most studies [11, 14, 16, 22], and the frequency of salt addition was used to indicate dietary sodium consumption. It was reported that the frequency of salt addition is linearly correlated with 24-hour urinary sodium levels [21]. We also investigated the relationship between potassium consumption and AF incident using 24-hour urinary potassium levels and vegetable and fruit consumption.

Our study investigated the relationship between the frequency of salt addition and AF incident and AF-related complications. Moreover, it demonstrates the effect of potassium consumption on AF incidents in a population with high sodium consumption.

## 2. Materials and Methods

### 2.1 Study Population

The UK Biobank is a population-based cohort study comprising >500,000 participants enrolled between 2006 and 2010. Participants underwent assessments at one of 22 England, Wales, and Scotland centers. Researchers assessed the UK Biobank data once the UK Biobank accepted the research proposal. All participants submitted signed informed consent. The previous report described the study design and methods in detail [23].

**Fig. 1.**
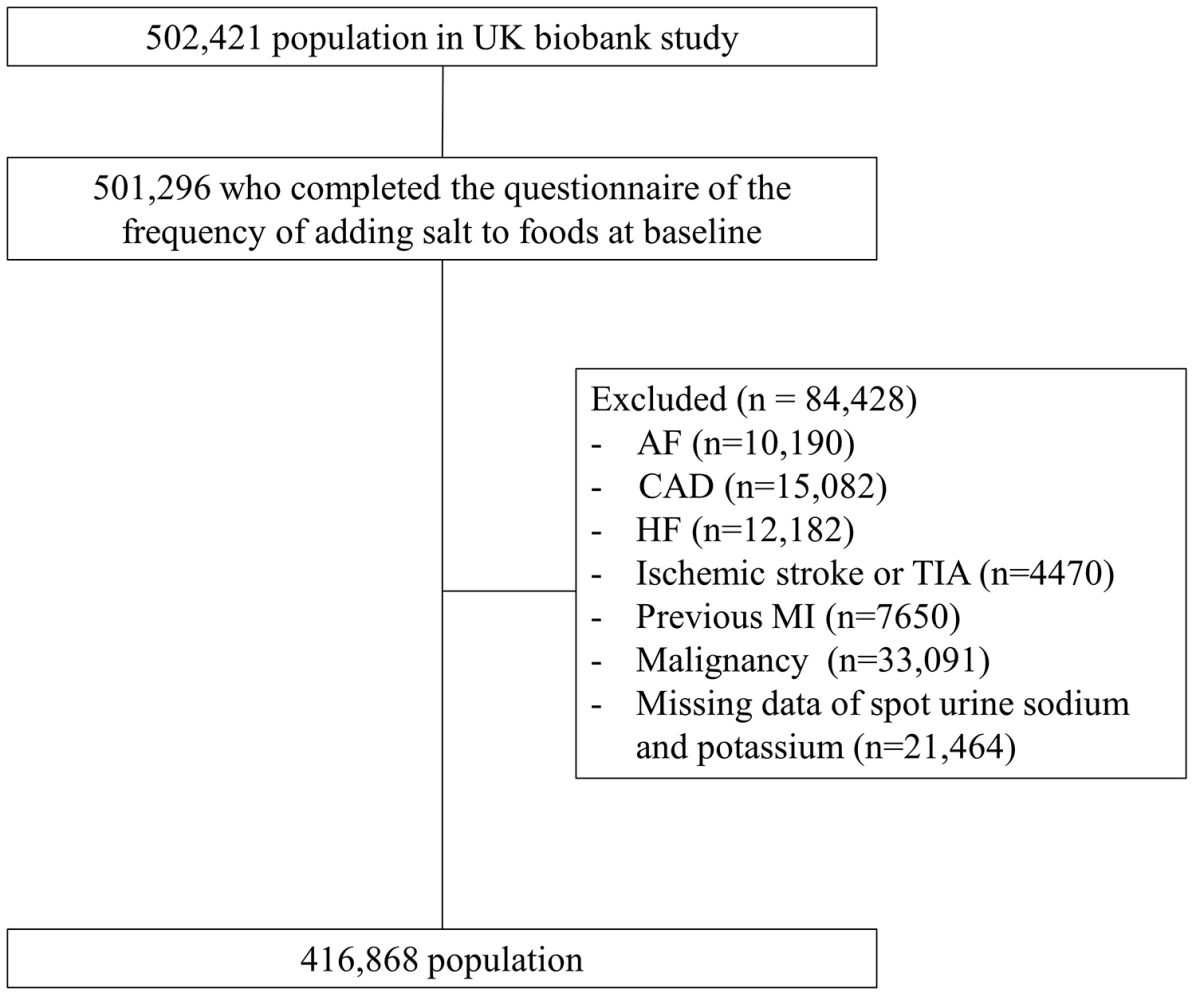
**Inclusion and exclusion processes from the UK biobank study**. Among 502,421 participants, 501,296 participants answered the questionnaire about the frequency of salt addition to foods. After excluding participants with previous AF, CAD, HF, ischemic stroke or TIA, MI, and malignancy and those without data of spot urine sodium and potassium, 416,868 participants were enrolled. Abbreviations: AF, atrial fibrillation; CAD, coronary artery disease; HF, heart failure; TIA, transient ischemic attack; MI, myocardial infarction.

We included participants who completed the dietary recall regarding the frequency of salt addition at the time of enrollment. We excluded individuals who had history of AF (n = 10,190), coronary artery disease (CAD) (n = 15,082), HF (n = 12,182), stroke or transient ischemic attack (TIA) (n = 4470), previous myocardial infarction (MI) (n = 7650), malignancy (n = 33,091), and those who had missing data of spot urine sodium and potassium (n = 21,464). Overall, this study included 416,868 participants (Fig. 1).

### 2.2 Assessment of Data

Participants completed the “Do you add salt to your foods?” questionnaire at enrollment. Participants chose one answer from five response options: (1) never/rarely; (2) sometimes; (3) usually; (4) always; (5) prefer not to answer. Participants who chose “prefer not to answer” were recorded as those with missing values.

Participants completed the questionnaire regarding the dietary assessment administered using the Qxford WebQ, which queries participants about the consumption of foods and drinks over the past 24 hr from 2009 to 2012 [24, 25]. Regular physical activity was ≥450 metabolic equivalent task (MET) minutes of moderate or vigorous weekly activity. The definition for moderate drinking was as follows: for men: >0 and ≤28 g/day; for women: >0 and ≤14 g/day [26]. Regarding vegetable consumption, participants reported the number of heaped tablespoons of salad/raw vegetables and cooked vegetables consumed per day or selected “less than one”, “do not know”, or “prefer not to answer”. Regarding fruit consumption, participants provided the number of fresh and dried fruit pieces per day or selected “less than one”, “do not know”, or “prefer not to answer”. One piece of fresh and two pieces of dried fruit were counted as a serving. Two heaped tablespoons of vegetables were also considered a serving [27].

Urine samples were collected when participants first visited. Sodium and potassium urine levels were measured using Beckman Coulter AU5400 (Beckman Coulter Inc, Brea, CA, USA) with ion-selective electrode analysis. The detailed method is available on the website [28]. Estimated 24-hour sodium and potassium levels were calculated from the measurement of spot sodium and potassium using the Kawasaki formulae [29].

### 2.3 Assessment of Covariates and Outcomes

Covariates and outcomes were defined using the International Classification of Disease, 10th Revision (ICD-10). AF was defined by ICD-10 code I48. Total CVD events were composed of stroke, systemic embolism, HF, and CAD. Incident CAD was composed of acute MI and death from CAD or MI. Incident acute MI was defined by ICD-10 codes I21-22; incident stroke was defined by ICD-10 codes I63-64; incident systemic embolism was defined by ICD-10 codes I74; the ICD-10 codes I110, I150, and I197.1 defined incident HF admission. Other covariates defined using ICD-10 codes are described in **Supplementary Table 1**.

### 2.4 Statistical Analysis

Student’s *t*-test was utilized for continuous variables presented as the mean ± standard deviation, while the Chi-square test was used for categorical variables, presented as proportions to compare the baseline characteristics. The incidence rates were computed by dividing the number of events by person-time at risk and the 95% confidence intervals (CIs) derived from exact Poisson distributions. To compare the incidence and risk of AF and CVD related to the frequency of slat addition, Cox proportional hazard models were employed. Assessment of the proportional hazard assumption was conducted using the Schoenfeld residuals. Potential confounding factors included sex, age, body mass index (BMI), regular physical activity, Townsend deprivation index, moderate drinking, smoking, dyslipidemia, hypertension, and chronic kidney disease (CKD). Statistical analyses were performed using R software version 4.1.0 (The R Foundation, https://www.r-project.org/). Statistical significance was considered at a *p*-value < 0.05.

## 3. Results

### 3.1 Baseline Characteristics

Table 1 summarizes the baseline characteristics of the study cohort in categories of the frequency of salt addition. Individuals who more frequently added salt to foods had higher BMI, waist, and Townsend deprivation index, a higher proportion of current smokers and moderate drinking, a lower proportion of hypertension, and less regular physical activity than those who added salt to foods less frequently. In addition, compared with individuals who added less salt to foods, vegetable and fruit consumption amounts were lower in individuals who more regularly added salt to foods. As the frequency of adding salt to food increased, the 24-hour sodium levels and total energy consumption increased.

**Table 1.** **Baseline characteristics based on the frequency of salt addition**.

	Never/rarely	Sometimes	Usually	Always	*p*-value
	(n = 231,427)	(n = 117,314)	(n = 48,186)	(n = 19,941)	
Age, years	56.5 ± 8.1	56.4 ± 8.1	56.9 ± 8.1	55.8 ± 8.3	<0.001
Male (%)	100,102 (43.3)	53,257 (45.4)	24,334 (50.5)	9510 (47.7)	<0.001
BMI, kg/m^2^	27.1 ± 4.7	27.5 ± 4.8	27.7 ± 4.7	27.9 ± 5.1	<0.001
Waist, cm	89.0 ± 13.1	90.4 ± 13.3	91.6 ± 13.4	92.0 ± 13.8	<0.001
Systolic BP, mmHg	138.1 ± 18.8	137.5 ± 18.5	137.8 ± 18.5	136.8 ± 18.7	<0.001
Diastolic BP, mmHg	82.4 ± 10.2	82.4 ± 10.1	82.5 ± 10.1	82.4 ± 10.3	0.524
Townsend deprivation index	–1.5 ± 3.0	–1.3 ± 3.1	–1.1 ± 3.2	–0.2 ± 3.5	<0.001
Current smoker (%)	18,544 (8.0)	13,258 (11.3)	7388 (15.4)	4715 (23.8)	<0.001
Moderate drinking (%)	32,611 (20.8)	19,897 (24.3)	10,531 (30.6)	4577 (36.2)	<0.001
Regular physical activity (%)	119,089 (51.5)	58,613 (50.0)	23,587 (48.9)	8928 (44.8)	<0.001
Hypertension (%)	62,545 (27.0)	28,677 (24.4)	11,436 (23.7)	4819 (24.2)	<0.001
Diabetes (%)	10,150 (4.4)	5253 (4.5)	2153 (4.5)	911 (4.6)	0.429
Dyslipidemia (%)	28,263 (12.2)	13,758 (11.7)	5872 (12.2)	2326 (11.7)	<0.001
ESRD or CKD (%)	1526 (0.7)	682 (0.6)	261 (0.5)	111 (0.6)	0.002
Estimated 24 hr sodium excretion (mg/day)	4039.7 ± 1184.6	4167.4 ± 1213.9	4276.4 ± 1248.8	4360.8 ± 1293.2	<0.001
Estimated 24 hr potassium excretion (mg/day)	2756.7 ± 586.4	2732.8 ± 590.7	2742.0 ± 591.0	2663.2 ± 594.2	<0.001
Vegetable and fruit (Svs/d)	5.3 ± 2.9	5.0 ± 2.9	4.8 ± 2.9	4.5 ± 3.3	<0.001
Total energy consumption (Kcal/day)	2094.0 ± 743.0	2147.4 ± 787.1	2180.1 ± 822.0	2184.3 ± 917.3	<0.001

Abbreviations: BMI, body mass index; BP, blood pressure; ESRD, end-stage renal disease; CKD, chronic kidney disease; hr, hours; Sys/d, daily servings.

### 3.2 Risk of AF Incident Based on the Frequency of Salt Addition

During the median follow-up period of 11.9 years (interquartile range: 11.2–12.6), 4.6% of participants were diagnosed with AF. The incidence of new-onset AF increased as the frequency of salt addition increased (Table 2). Among groups who never or rarely, sometimes, usually, and always added salt to foods, the incidences of new-onset AF were 3.83, 3.97, 4.55, and 4.72, respectively.

**Table 2.** **Incidence of new-onset AF based on the frequency of salt addition**.

	Never/rarely	Sometimes	Usually	Always
	(n = 231,427)	(n = 117,314)	(n = 48,186)	(n = 19,941)
Case	10,216	5364	2513	1071
Incidence per 1000 person-years	3.83	3.97	4.55	4.72
Absolute rate difference per 1000 person-years (95% CI)	1 (reference)	0.14 (0.01–0.27)	0.72 (0.54–0.90)	0.90 (0.63–1.16)

Abbreviations: AF, atrial fibrillation; CI, confidence interval.

Table 3 summarizes the AF incident risk based on the salt addition frequency. After adjusting for sex and age, compared with individuals who never or rarely added salt to foods, the risks of AF incident were increased by 3% (hazard ratio [HR] 1.03; 95% CI 1.00–1.07) in those who sometimes added salt, by 8% (HR 1.08; 95% CI 1.03–1.13) in those who usually added salt, and by 25% (HR 1.25; 95% CI 1.18–1.34) in those who always added salt (Table 3). After adjusting for potentially confounding clinical covariates such as age, sex, BMI, regular physical activity, Townsend deprivation index, moderate drinking, smoking, dyslipidemia, hypertension, and CKD, individuals who always added salt were significantly associated with a higher risk of AF incident (HR 1.15; 95% CI 1.06–1.24). Even after additional adjustments of dietary (vegetable and fruit) and total energy consumption, this association remained (HR 1.37; 95% CI 1.08–1.73) compared to those who never or rarely added salt to foods.

**Table 3.** **Risk for AF incident based on the frequency of salt addition**.

	Never/rarely	Sometimes	Usually	Always
	(n = 231,427)	(n = 117,314)	(n = 48,186)	(n = 19,941)
Sex and age adjusted HR (95% CI)	1 (reference)	1.03 (1.00–1.07)	1.08 (1.03–1.13)	1.25 (1.18–1.34)
Multivariable* adjusted HR (95% CI)	1 (reference)	1.03 (0.99–1.07)	1.06 (1.00–1.11)	1.15 (1.06–1.24)
Multivariable* + dietary consumption adjusted HR † (95% CI)	1 (reference)	1.04 (1.00–1.08)	1.05 (1.00–1.12)	1.16 (1.07–1.25)
Multivariable* + total energy consumption adjusted HR ‡ (95% CI)	1 (reference)	1.16 (1.04–1.30)	1.15 (0.99–1.33)	1.37 (1.08–1.73)

Abbreviations: AF, atrial fibrillation; HR, hazard ratio; CI, confidence interval; BMI, body mass index; CKD, chronic kidney disease. * Adjusted for age, sex, BMI, Townsend deprivation index, regular physical activity, smoking, moderate drinking, hypertension, dyslipidemia, diabetes, and CKD. † Vegetable consumption and fruit consumption. ‡ A total of 41,285 participants were available.

In the subgroup analysis for white participants, the incidence and risk of new-onset AF increased with the higher frequency of salt addition (**Supplementary Tables 2,3**). After further adjustment for consumption of vegetables and fruit or total energy consumption, the effect of the higher frequency of salt addition on the risk of AF incident remained the same.

**Fig. 2.**
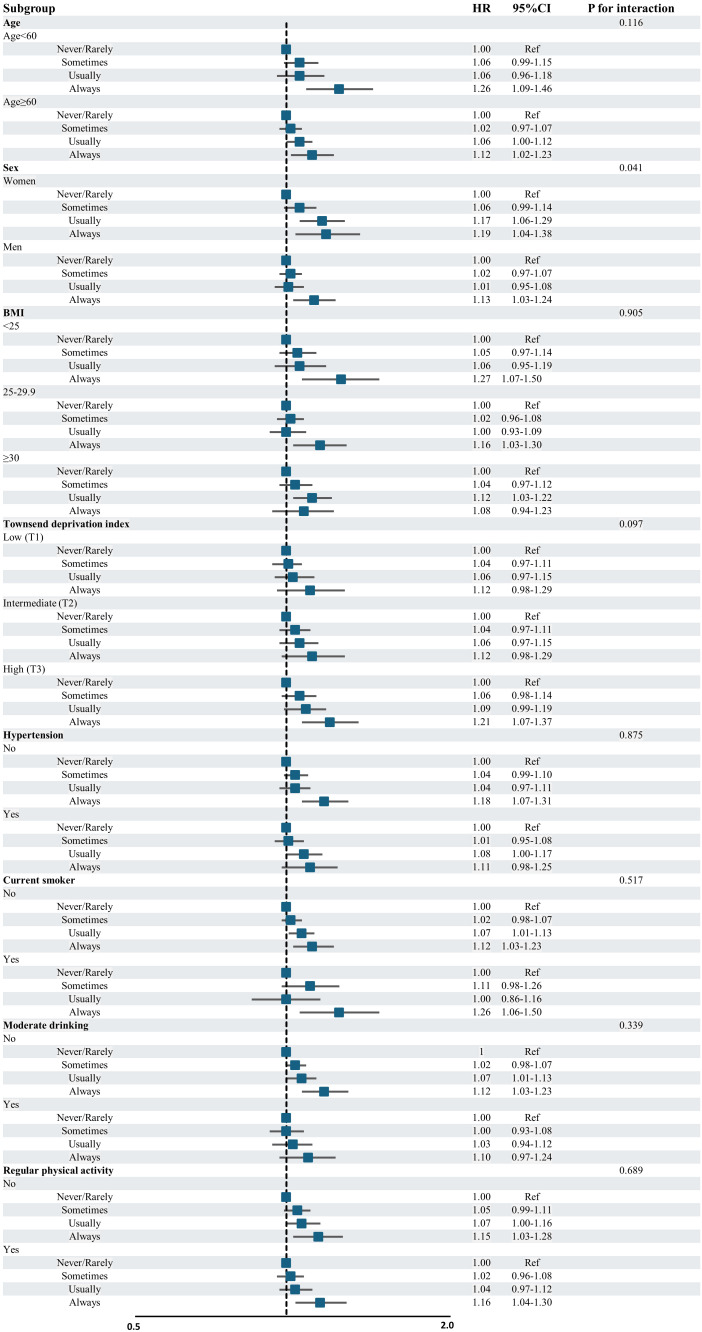
**Stratified analysis for AF incident risk based on the frequency of salt addition**. Fig. 2 displays the association between the risk of AF incident and frequency of salt addition in subgroup analysis. It demonstrates that participants who added salt to foods more frequently had an increased risk of AF incidents regardless of age, BMI, economic status, comorbidities, and lifestyle, except for sex. Abbreviations: AF, atrial fibrillation; HR, hazard ratio; CI, confidence interval; Ref, reference; BMI, body mass index.

Fig. 2 summarizes the stratified analysis based on potential risk factors. In the stratified analysis, the risk of incident AF increased with the higher frequency of salt addition group regardless of age, BMI, economic status, comorbidities, and lifestyle. The risk of AF incidents increased with the higher frequency of salt addition group among women than men (*p* for interaction = 0.041).

### 3.3 Relationship between Salt Consumption and Urinary Potassium or Vegetable and Fruit Consumption

**Fig. 3.**
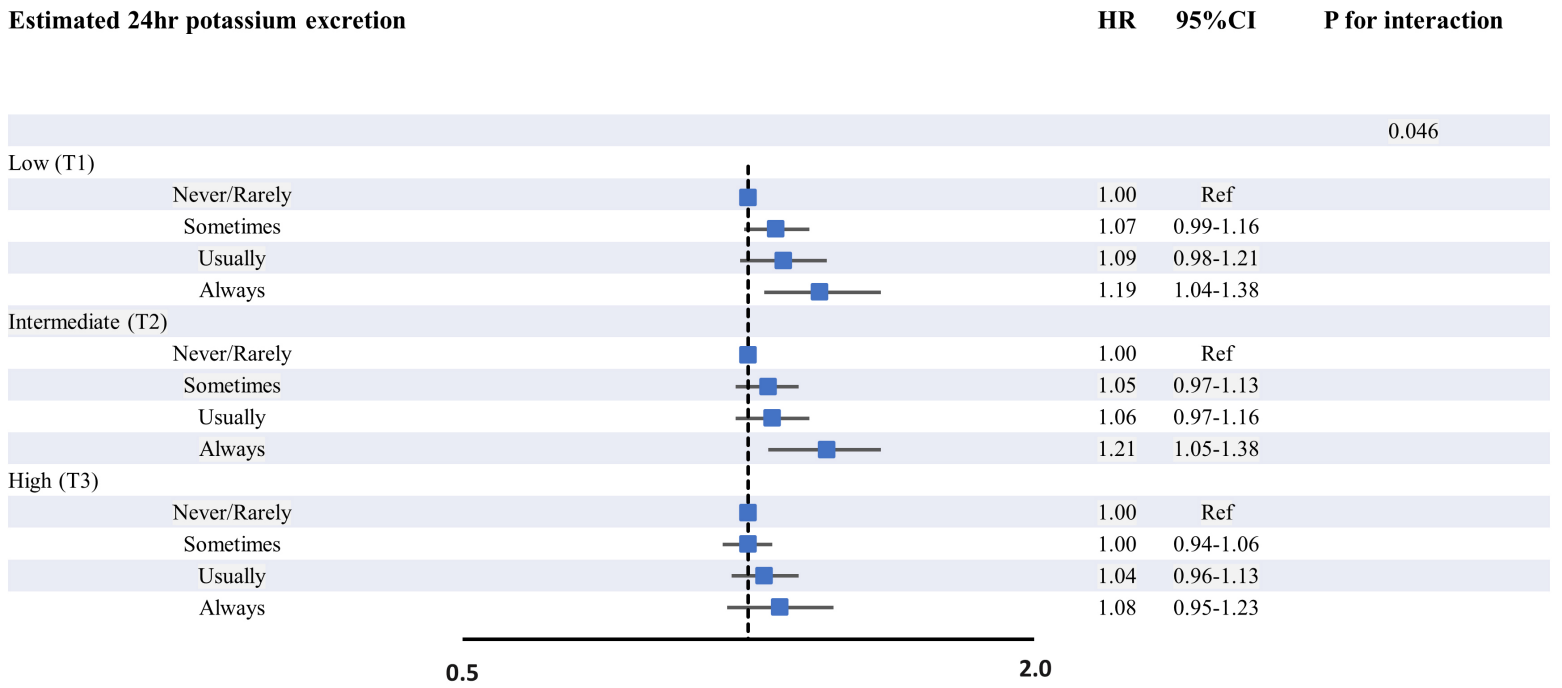
**Association between the frequency of salt addition and urinary potassium in relation to risk of AF incident**. In Fig. 3, we divided the estimated 24-hour potassium excretion into tertiles and analyzed the association between the frequency of salt addition and the risk of incident AF in each group. It illustrates that the risk of AF incidents increased in participants who always added salt to foods, especially in low and intermediate-potassium level groups. Abbreviations: AF, atrial fibrillation; HR, hazard ratio; CI, confidence interval; Ref, reference.

The relationship between the frequency of salt addition and urinary potassium with the risk of AF incident is depicted in Fig. 3. The risk of AF incident was higher in the group that always added salt to foods compared to the control group among individuals with low urinary potassium levels (HR 1.19; 95% CI 1.04–1.38) and intermediate urinary potassium levels (HR 1.21; 95% CI 1.05–1.38). The risk of AF incident increased significantly in those with low or intermediate urinary potassium levels than in those with high levels (*p *for interaction = 0.046; Fig. 3). Although there was no statistical significance in the vegetable and fruit group, the risk of AF incident showed a tendency to increase in the low and intermediate groups compared to the high group (**Supplementary Fig. 1**).

### 3.4 Risk of Mortality, Stroke, and HF in Patients with AF

**Fig. 4.**
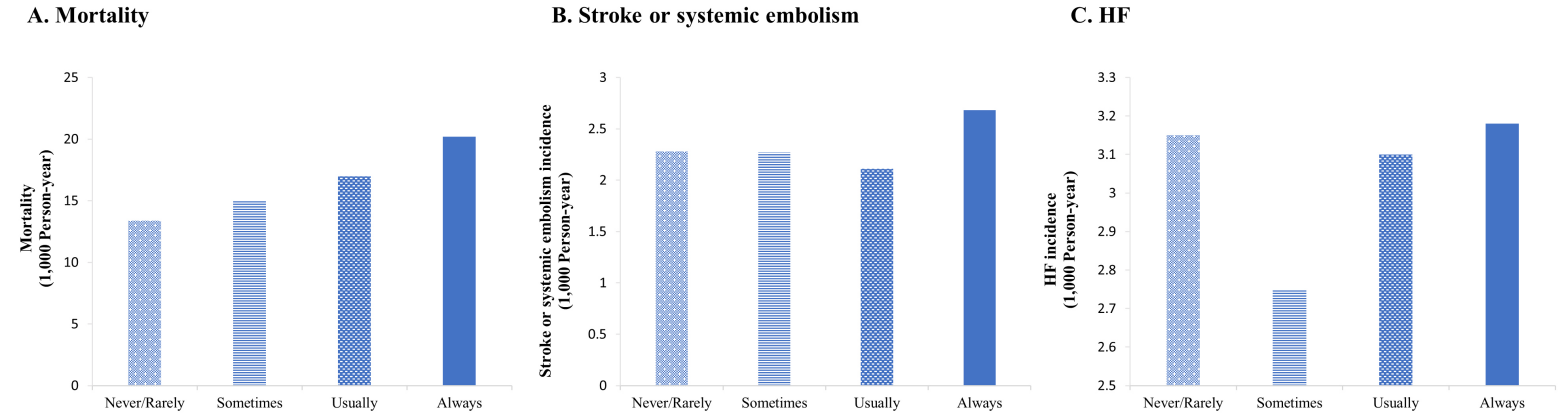
**The incidence of stroke or systemic embolism, HF, and mortality based on the frequency of salt addition in AF patients**. (A) Mortality. (B) Stroke or systemic embolism. (C) HF. Fig. 4 shows the association between the frequency of salt addition and the incidence of mortality, stroke or systemic embolism, and HF in patients with AF. (A) shows that the incidence of mortality increased in patients with a higher frequency of salt addition. (B) shows that the incidence of stroke or systemic embolism was highest in patients who always added salt to foods. (C) shows that the incidence of HF was highest in patients who always added salt to foods. Abbreviations: AF, atrial fibrillation; HF, heart failure.

Among 19,164 participants who were diagnosed with AF, the incidence of all-cause mortality elevated dramatically with the higher frequency of salt addition (Fig. 4A). Incidence of stroke or systemic embolism and HF did not vary significantly based on the frequency of salt addition. However, the incidence of stroke or systemic embolism and HF was higher in the group that always added salt to foods compared with other frequencies of salt addition (Fig. 4B,C).

## 4. Discussion

The present study, conducted with individuals from the UK Biobank, revealed a compelling relationship between a higher frequency of salt addition and a substantially heightened risk of AF incident, even after adjusting for multiple variables. In addition, our findings showed the same relationship between a higher frequency of salt addition and risk of AF incidents, even in the white population group. High potassium consumption reduced the risk of AF incident in the high salt consumption group compared to low potassium consumption. Furthermore, within the subgroup of participants diagnosed with AF, a gradually elevated incidence of all-cause mortality was observed based on the frequency of salt addition.

### 4.1 The Association between Sodium Consumption and Risk of New-Onset AF

Several studies showed conflicting results about the correlation between sodium consumption and the risk of new-onset AF. In another study, compared with low sodium consumption, high sodium consumption increased the risk of new-onset AF [20]. This study used a seven-day food recall to evaluate dietary sodium consumption. A further study demonstrated a U-shaped correlation between sodium consumption and the risk of incident AF [19]. This study used 24-hour urinary sodium levels based on spot urine sodium measurements to indicate dietary sodium consumption. In our study, the frequency of salt addition was used as a marker for dietary sodium consumption. Although the frequency of salt addition may not offer precise quantitative details on total sodium consumption, it could serve as a surrogate marker to indicate an individual’s preference for sodium consumption [30]. Moreover, as in another study [21], our study showed a correlation between the frequency of salt addition and 24-hour urinary sodium levels.

### 4.2 Mechanism of Increased AF by High Sodium Consumption

High sodium consumption is a significant factor leading to increased blood pressure [10] and is related to an increased risk of CVD [16]. Both are recognized as risk factors for the development of AF. These factors may have influenced the higher risk of AF incidents associated with high sodium consumption. However, in the subgroup analysis of our study, high sodium consumption increased the risk of AF incident even in the group without hypertension, suggesting the involvement of other contributing factors. Another potential mechanism to consider is the impact of sodium-induced QT interval prolongation on the development of AF. In the meta-analysis study, the prolonged QTc interval is related to an increased risk of AF [31]. One study showed that high sodium consumption increased the QTc interval [32], explaining a possible mechanism for the effect of high sodium consumption on the development of AF.

### 4.3 Conflicting Effect of Sodium and Potassium

Several studies have shown conflicting impacts of sodium and potassium on health outcomes [22, 33, 34]. A recent study indicated that increased potassium consumption was related to decreased CVD risk, while increased sodium consumption was related to increased CVD risk [16]. However, the relationship between high potassium consumption and the risk of new-onset AF has yet to be established. In our study, we used estimated 24-hour potassium levels and the consumption of vegetables and fruits as indicators of potassium consumption to evaluate the relationship between potassium consumption and the risk of new-onset AF. In the high potassium excretion group, the effect of high sodium consumption on the risk of AF incident was attenuated. Therefore, it is postulated that high potassium consumption may influence the AF incident risk. However, the *p*-value for interaction was not statistically significant for vegetable and fruit consumption, suggesting the need for further research to identify a more accurate indicator for measuring dietary potassium consumption.

### 4.4 The Association between Sodium Consumption and AF-Related Outcomes in Patients with AF

Several studies showed that AF is related to an elevated risk of stroke, HF, and mortality [2, 5], and several scoring systems have been used to predict stroke in patients with AF [35, 36]. The correlation between increased salt consumption and the risk of CVD and HF has also been demonstrated in previous studies [12, 37, 38]. However, a relationship between sodium consumption and AF-related outcomes has yet to be established. In this study, we evaluated the association between sodium consumption and stroke or systemic embolism, HF, or all-cause mortality in AF patients. We observed a correlation between increased all-cause mortality and the frequency of salt addition. We found no correlation between increasing sodium consumption and the incidence of stroke or systemic embolism in the AF patient group. However, their incidence was the highest in the group of always adding salt to foods compared to other groups. While additional research is needed, this study shows that high salt consumption in patients with AF may be associated with a significantly increased incidence of AF-related outcomes.

### 4.5 Study Limitations

There are some limitations to this study. In our research and previous studies, the frequency of salt addition may indicate dietary sodium consumption. However, it may not accurately measure total sodium consumption. This limitation suggests a potential gap in understanding the comprehensive impact of sodium consumption on AF incidents. Another limitation could be the potential impact of dietary variations across different racial groups on the outcomes. In our study, we conducted additional subgroup analysis focusing on the Caucasian population, and our findings confirmed consistent results regarding the association between sodium consumption and AF incidence. Since this was an observational study, it may still have unmeasured or residual confounders. Thus, additional research, such as randomized trials, is needed to investigate this matter further.

## 5. Conclusions

This study demonstrates an association between the frequency of salt addition to foods and the risk of AF incidents. Moreover, higher sodium consumption significantly increased the risk of AF incidents, particularly in participants with low urinary potassium levels. Furthermore, in patients already diagnosed with AF, increased salt consumption correlated with higher all-cause mortality. These findings underscore the importance of dietary modifications, particularly reducing sodium and increasing potassium consumption, in managing and potentially reducing the risk of AF. However, further research and randomized clinical trials are required to confirm these findings and offer a more comprehensive understanding of the association between sodium and potassium consumption and AF.

## Availability of Data and Materials

The original contributions presented in the study are included in the article; further inquiries can be directed to the corresponding author.

## Supplementary Material

Supplementary material associated with this article can be found, in the online version, at https://doi.org/10.31083/j.rcm2509332.
