# Supplementary material for: Association of Adding Salt to Foods and Potassium Intake with Incident Atrial Fibrillation in the UK Biobank Study
Source: Rev Cardiovasc Med. 2024 Sep 19;25(9):332. doi: 10.31083/j.rcm2509332 (PMC11440396; doi:10.31083/j.rcm2509332)

Supplementary Table 1. Definitions and ICD-10 codes used for defining the comorbidities and clinical outcomes.

Comorbidities	Definitions	ICD-10 codes or conditions
AF	Defined from diagnosis*	ICD-10: I48
HF	Defined from diagnosis*	ICD-10: I11.0, I50, I97.1
Hypertension	Defined from diagnosis*	ICD-10: I10, I11, I12, I13, I15 and antihypertensive medication
Diabetes mellitus	Defined from diagnosis* plus treatment	ICD-10: E10, E11, E12, E13, E14<br>Treatment: all kinds of oral antidiabetics and insulin.
Dyslipidemia	Defined from diagnosis*	ICD-10: E78
Ischemic stroke	Defined from diagnosis*	ICD-10: I63, I64
TIA	Defined from diagnosis*	ICD-10: G45
MI	Defined from diagnosis*	ICD-10: I21, I22, I25.2
CKD	Defined from eGFR or diagnosis*<br>(if laboratory value was not available, diagnosis code was used)	eGFR <60mL/min per 1.73 m^2<br>ICD-10: N18, N19
ESRD	Defined from national registry for severe illness.	Patients with end-stage renal disease undergoing chronic dialysis or received a kidney transplant.
Malignancy	Defined from diagnoses of cancer (non-benign)	ICD-10: C00-C97

ICD-10, International Classification of Diseases-10th Revision; AF, atrial fibrillation; HF, heart failure; TIA, transient ischemic attack; MI, myocardial infarction; CKD, chronic kidney disease; eGFR, estimated glomerular filtration rate; ESRD, end-stage renal disease.

*To ensure accuracy, comorbidities were established based on one inpatient or two outpatient records of ICD-10 codes in the database.

Supplementary Table 2. Incidence of new-onset AF based on the frequency of salt addition in white participants.

	Never/Rarely (n=208347)	Sometimes (n=102226)	Usually (n=41407)	Always (n=15823)
Case	9437	4892	2290	934
Incidence Per 10,000 Person-Years	39.2	41.5	48.2	51.9
Absolute rate difference Per 10,000 Person-Years (95% CI)	1 (reference)	2.3 (0.9-3.6)	9.0 (7.0-11.0)	12.6 (9.6-15.7)

AF, atrial fibrillation; CI, confidence interval.

Supplementary Table 3. Risk for incident AF based on the frequency of salt addition in white participants.

	Never/Rarely (n=208347)	Sometimes (n=102226)	Usually (n=41407)	Always (n=15823)
Sex and age adjusted HR (95% CI)	1 (reference)	1.04 (1.01-1.08)	1.09 (1.04-1.14)	1.27 (1.19-1.36)
Multivariable* adjusted HR (95% CI)	1 (reference)	1.03 (0.99-1.08)	1.06 (1.00-1.12)	1.15 (1.06-1.25)
Multivariable* + dietary consumption adjusted HR † (95% CI)	1 (reference)	1.04 (1.00-1.08)	1.06 (1.00-1.12)	1.16 (1.07-1.27)
Multivariable* + total energy consumption adjusted HR ‡ (95% CI)	1 (reference)	1.15 (1.02-1.29)	1.16 (1.00-1.36)	1.35 (1.05-1.74)

AF, atrial fibrillation; HR, hazard ratio; CI, confidence interval; BMI, body mass index, CKD, chronic kidney disease.

* Adjusted for age, sex, BMI, Townsend deprivation index, smoking, moderate drinking, regular physical activity, hypertension, dyslipidemia, diabetes and CKD.

† Vegetable consumption and fruit consumption.

‡ A total of 36949 participants were available.

Supplementary Fig. 1. Association between the frequency of salt addition and vegetable and fruit consumption in relation to risk of incident AF. Supplementary Fig. 1 displays that the risk of incident AF tended to increase in the low and intermediate group compared to the high group, although there was no statistical significance. AF, atrial fibrillation; CI, confidence interval.

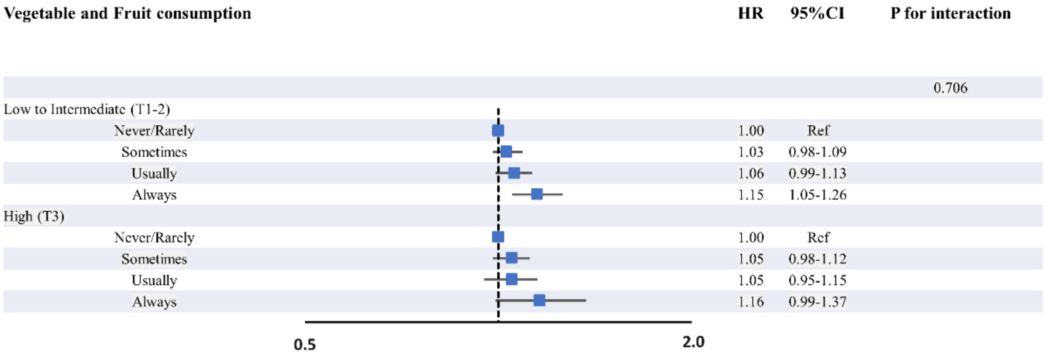

Supplement: Supplementary file 1 [file 2153-8174-25-9-332-s1.pdf]
